# Neighborhood Poverty Exposure in Adulthood and Cognitive Outcomes in Later Life

**DOI:** 10.1002/alz70860_103557

**Published:** 2025-12-23

**Authors:** Lucie Kalousova

**Affiliations:** ^1^ Vanderbilt University, Nashville, TN, USA

## Abstract

**Background:**

Neighborhood poverty is a well‐documented social determinant of health, yet its association with dementia risk over the life course remains understudied. This study examines whether exposure to neighborhood poverty at different life course stages predicts dementia risk and evaluates the cumulative and time‐specific effects of poverty exposure using sequence analysis and regression modeling.

**Method:**

Using data from the PSID analytic sample, we applied sequence analysis to classify individuals into six distinct poverty trajectory types from 1968 to 2021. Logistic regression models with odds ratios and marginal structural models with inverse probability weighting were used to estimate the associations between neighborhood poverty exposure and dementia risk. Life course periods were defined as young adulthood (ages 18–29), mid‐adulthood (ages 30–49), and later adulthood (ages 50 and older).

**Result:**

Neighborhood poverty trajectories are significantly associated with dementia risk. Participants in trajectories characterized by high or fluctuating poverty exposure (Types 3 and 5) exhibit the highest odds of a positive dementia screener, with odds ratios exceeding 2.0 in unadjusted models. Exposure to neighborhood poverty in young adulthood shows the largest impact, with an odds ratio of 1.24 in fully adjusted models accounting for mediation. Later adulthood poverty exposure is not significantly associated with dementia risk after adjusting for earlier exposures. Predicted probabilities highlight substantial variability across trajectories, with a 14 percentage‐point difference in dementia risk between consistently low‐ and high‐poverty trajectories.

**Conclusion:**

Cumulative and early‐life exposure to neighborhood poverty significantly increases dementia risk, supporting the early‐life hypothesis. These findings underscore the importance of addressing structural inequalities early in life to reduce cognitive health disparities later in life.

